# Greater Left Inferior Frontal Activation for SVO than VOS during Sentence Comprehension in Kaqchikel

**DOI:** 10.3389/fpsyg.2016.01541

**Published:** 2016-10-14

**Authors:** Masatoshi Koizumi, Jungho Kim

**Affiliations:** ^1^Department of Linguistics, Graduate School of Arts and Letters, Tohoku UniversitySendai, Japan; ^2^Department of Foreign Languages, Kyoto Women’s UniversityKyoto, Japan

**Keywords:** basic word order, Guatemala, left inferior frontal gyrus, processing load, syntactic complexity

## Abstract

Cortical activations during the processing of Kaqchikel transitive sentences with canonical and non-canonical word orders were investigated using functional magnetic resonance imaging. Kaqchikel is an endangered Mayan language spoken in Guatemala. The word order in this language is relatively flexible. We observed higher cortical activations in the left inferior frontal gyrus for sentences with the subject-verb-object (SVO) word order, as compared to sentences with the verb-object-subject (VOS) word order, suggesting that Kaqchikel sentences are easier to process when they have the VOS order than when they have the SVO order. This supports the traditional analysis of Mayan word order: the syntactically simplest word order of transitive sentences in Mayan languages, including Kaqchikel, is VOS. More importantly, the results revealed that the subject-before-object word order preference in sentence comprehension, previously observed in other languages, might not reflect a universal aspect of human languages. Rather, processing preference may be language-specific to some extent, reflecting syntactic differences in individual languages.

## Introduction

It has been reported that in many flexible word order languages, sentences with a transitive verb (V) induce a lower processing load when the subject (S) precedes the object (O) (SO WORD ORDER = SOV, SVO, VSO) than when the opposite occurs (OS WORD ORDER = OSV, OVS, VOS). For instance, behavioral studies have found that OSV sentences take longer to process than SOV sentences in Japanese (Mazuka et al., 2002; Tamaoka et al., 2005). Similar results have been reported for many other languages (see, for example, Sekerina, 1997 for Russian; Kaiser and Trueswell, 2004 for Finnish; Tamaoka et al., 2011 for Sinhalese; Kim, 2012 for Korean). From a neurophysiological viewpoint, studies with functional magnetic resonance imaging (fMRI) have reported greater activations of the left inferior frontal gyrus (left IFG) during the processing of OS sentences compared to SO sentences (Grewe et al., 2007 for German; Kinno et al., 2008 and Kim et al., 2009 for Japanese). Event-related potentials (ERP) research has also found that OS orders, relative to SO orders, elicit a (sustained) anterior negativity and/or P600, suggesting that processing OS word orders incurs a larger working memory load (Roesler et al., 1998 for German; Ueno and Kluender, 2003 and Hagiwara et al., 2007 for Japanese; Erdocia et al., 2009 for Basque).

Thus, there is solid evidence that subject-before-object word orders are easier to process than the object-before-subject word orders in many world languages. Following Koizumi et al. (2014), we refer to this generalization as “the SO word order preference in sentence comprehension” in this paper. This observation raises two related questions: (i) why should this be the case? and (ii) is this preference universal across all human languages? In the psycholinguistic literature, there are two major broad theoretical positions regarding the possible factors affecting word order preference in sentence processing (Koizumi et al., 2014). One view, which Koizumi et al. (2014) refer to as INDIVIDUAL GRAMMAR THEORY, posits that the SO word order preference is primarily due to grammatical factors in each language, such as syntactic complexity. According to many sentence-processing theories, other things being equal, sentences with the syntactically determined basic word order of a language have a lower processing load than those with other grammatically possible word orders with additional filler-gap dependencies in the language (Pritchett and Whitman, 1995; O’Grady, 1997; Gibson, 2000; Hawkins, 2004; Marantz, 2005). Thus, from the perspective of individual grammar theory, SO word orders were found to be preferred because they were syntactically simpler than other orders in the languages under study.

In contrast, what may be called UNIVERSAL COGNITION THEORY hypothesizes that word order preferences are largely attributable to universal human cognitive features (e.g., conceptual accessibility), so that SO word orders should be preferred regardless of the basic word order of any individual language (Bornkessel-Schlesewsky and Schlesewsky, 2009a,b; Tanaka et al., 2011; Kemmerer, 2012). The fact that a vast majority of the world’s languages have one of the SO word orders as their basic word order (SOV 48%, SVO 41%, VSO 8%, VOS 2%, OVS 1%, and OSV 0.5%, according to Dryer, 2013; see also Gell-Mann and Ruhlen, 2011) strongly suggests that there may be such universal features. Furthermore, various studies have shown that prominent entities with properties such as agency, animacy, concreteness, and so on, tend to appear sentence-initially as subjects (Slobin and Bever, 1982; Bock and Warren, 1985; Hirsh-Pasek and Golinkoff, 1996; Primus, 1999; Branigan et al., 2008; Bornkessel-Schlesewsky and Schlesewsky, 2009a,b; Tanaka et al., 2011). Given these, Kemmerer (2012) suggests that the SO word order preference reflects the most natural way of linearizing and nesting the core conceptual components of actions in Broca’s area. Universal cognition theory, therefore, leads to the expectation that SO orders should be easier to process than OS orders, regardless of the syntactic nature of any individual language.

A third possibility, which is referred to as USAGE-BASED THEORY in this paper, concerns production frequency. It has been demonstrated that frequency sometimes has a strong influence on processing cost (e.g., Trueswell et al., 1993). That is, speakers of the language learn how to process sentences efficiently based on their experiences, and frequent words and constructions tend to be processed with greater speed and a higher level of accuracy than infrequent expressions. Thus, it may be the case that OS word orders were more difficult to process than SO word orders in previous studies because of the lower frequencies of the former in the languages under examination.

Note that these three kinds of factors are not mutually exclusive. There is ample evidence that they all affect human sentence processing in one way or another, as demonstrated in numerous studies, such as those mentioned above. What has not been clear is their relative contribution or strength. All three theories/factors correctly account for the SO word order preference in sentence comprehension in SO languages. In Japanese, for example, SOV is easier to process than OSV, as alluded to above. This may be because SOV is (1) the syntactically basic word order (individual grammar theory), (2) an SO order (universal cognition theory), and/or (3) more frequent than OSV (usage-based theory). Thus, it is difficult, if not impossible, to evaluate the relative strengths of these factors solely focusing on SO languages such as Japanese. To determine which is the primary factor for the observed word order preference, it is necessary to study languages for which the three kinds of factors would create different predictions. To this end, Koizumi et al. (2014) conducted a sentence processing experiment in Kaqchikel. Kaqchikel is a Mayan language spoken in Guatemala and listed as an endangered language in the *UNESCO Atlas of the World’s Languages in Danger* (Moseley, 2010). The word order in Kaqchikel is relatively flexible. Although its syntactically basic word order is VOS, SVO is more frequently used. In Koizumi et al.’s (2014) experiment, transitive sentences, either semantically plausible or implausible, in three different word orders (i.e., VOS, VSO, and SVO), as well as filler sentences, were aurally presented in a random order to the participants through headphones. The participants were asked to judge whether each sentence was semantically plausible (correct) or not and to push the YES button (correct sentence) or NO button (incorrect sentence) as quickly and accurately as possible according to their judgment. The reaction time from the beginning of each stimulus sentence until the button was pressed was measured. Researchers found that semantically plausible sentences in VOS order were processed faster than those in SVO or VSO order. This suggests that in Kaqchikel, VOS, an OS order, is easier to process than SVO and VSO, both of which are SO orders, despite the higher production frequency of SVO. Based on these results, Koizumi et al. (2014) concluded that the SO order preference in sentence comprehension may not be universal; rather, processing load in sentence comprehension may be greatly affected by the syntactic nature of the individual language. The test items used in Koizumi et al. (2014) were sentences with an animate subject and an inanimate object (i.e., nonreversible sentences), but the results were replicated with sentences with an animate subject and object (i.e., reversible sentences), as reported in Kiyama et al. (2013).

Here we extend these two behavioral studies by examining cortical activations during the processing of Kaqchikel sentences with VOS and SVO orders. In particular, we investigate whether or not the left IFG universally favors SO orders to OS orders, as suggested by Kemmerer (2012) and others.

## Kaqchikel

Kaqchikel is morphologically ergative and head-marking [i.e., subjects and objects are not morphologically case-marked, and persons (first, second, or third) and numbers (singular or plural) of both subjects and objects are specified on the predicate]. Like many other Mayan languages, Kaqchikel allows different grammatical word orders. However, its syntactically basic word order is VOS, in which neither the subject nor object is topicalized or focused (Rodríguez Guaján, 1994; García Matzar et al., 1997; Tichoc et al., 2000; Ajsivinac Sian et al., 2004). VOS is thus typically used in a neutral context. An example of a VOS sentence is shown in (1) (CP [completive], ABS [absolutive], ERG [ergative], DET [determiner], 3 [third person], sg [singular], pl [plural], PM [plural marker]).

**Table:** 

(1)	*X-Ø-u-chöy*	*ri*	*chäj*	*ri*	*ajanel.* [VOS]
	CP-ABS3sg-ERG3sg-cut	DET	pine. tree	DET	carpenter
	‘The carpenter cut the pine tree.’			(Koizumi et al., 2014)	

When the subject is preposed before the verb, the subject tends to be interpreted as a topic, as illustrated in (2) (García Matzar et al., 1997).

**Table:** 

(2)	Ri	ajanel	x-Ø-u-chöy	ri	chäj. [SVO]
	DET	carpenter	CP-ABS3sg-ERG3sg-cut	DET	pine.tree
	‘The carpenter cut the pine tree.’			(Koizumi et al., 2014)	

Thus, SVO is pragmatically and syntactically marked.

There are two major analyses of Mayan word order and syntactic structure. One is a right specifier analysis a la Aissen (1992), according to which VOS is base-generated. The other is a predicate fronting analysis along the lines of Coon (2010), which says that VOS results from an obligatory movement of a predicate phrase (VP or some larger phrase). In either analysis, the derivation of SVO involves an additional movement of the subject. This is schematically shown in (3).

**Table:** 

(3)		Right Specifier Analysis	Predicate Fronting Analysis
	VOS:	[VOS]	[[t_i_ V O]_j_ [S_i_ t_j_]]
	SVO:	[S_i_ [VO t_i_]]	[S_i_ [[t_i_ V O]_j_ [t_i_’ t_j_]]]

Although precise syntactic structures of Kaqchikel are still under debate, for the purpose of this paper, it is sufficient to assume that VOS is structurally simpler than SVO (cf. England, 1991; Tada, 1993; Koizumi et al., 2014; Yasunaga et al., 2015).

It is interesting to note at this point that even though Kaqchikel’s syntactically basic word order is VOS, SVO is the most frequently used word order in this language (England, 1991; Rodríguez Guaján, 1994; Maxwell and Little, 2006; Kubo et al., 2015). Kubo et al. (2015), for example, reported that in their picture description experiment, the production frequency of SVO sentences was several times higher than that of VOS sentences (74.4% and 24.2%, respectively, of the total sentences produced). The reason SVO is most frequently produced may at least partially be that the topicalized subject produces cohesion in discourse (Koizumi et al., 2014). In fact, SVO appears more frequently than other word orders in many Mayan languages. Hence, it has been suggested that when examining the “basic word order” of Mayan languages, “syntactically determined basic word order” needs to be distinguished from “pragmatically determined basic word order” (Brody, 1984; England, 1991). We will come back to the issue of this discrepancy in the section “Discussion.”

## Materials and Methods

### Participants

Seventeen right-handed, healthy Kaqchikel native speakers with normal hearing participated in the experiment. However, only 16 participants (9 females) were included in the data analysis (mean age ± SD = 34.7 years ± 7.8). One participant was excluded from the final analysis because of technical problems. Two participants took part in only Session 1 because of poor health. Handedness was evaluated using the Edinburgh Handedness Inventory (Oldfield, 1971). All participants were living in Guatemala when they traveled to Japan to participate in the larger research project, of which the present experiment is a part. Before the experiment, all participants were provided with a sufficient explanation of the entire experiment and its safety, in accordance with the guidelines of Tohoku University. Written informed consent was obtained from each participant. Only those participants who gave written informed consent took part in the actual experiment. Approval for the study was obtained from the Ethics Committee of the Graduate School of Arts and Letters, Tohoku University.

### Stimuli

Semantically natural, grammatical, transitive sentences (i.e., “correct sentences”) were arranged into each of the two word orders (VOS and SVO), as shown in **Table 1**. Fifty-two pairs for 104 target sentences were created in this way. Additionally, 24 pairs for 48 sentences, which were grammatical but not semantically natural (i.e., “incorrect sentences”), were arranged in each of the two word orders. In order to morpho-syntactically differentiate the two argument roles, half of the sentences contained a singular subject and plural object, whereas the other half contained a plural subject and singular object. All 76 sentence pairs, consisting of 152 sentences, were counterbalanced and then categorized into two groups according to word order. All the stimulus sentences were recorded (32 bit, 11025 Hz) by a male native Kaqchikel speaker and saved as WAV sound files.

**Table 1:** Example sentences.

Condition	Example sentence							
A. VOS	a:	X-e-ru-pïs		ri	taq	lej	ri	ch‘utitata’
		CP-ABS3pl-ERG3sg-wrap		DET	PM	tortilla	DET	uncle
		[Correct sentence] “The uncle wrapped the tortillas.”						
	b:	x-e-ru-k’ät		ri	taq	nuq’u’	ri	üs
		CP-ABS3pl-ERG3sg-burn		DET	PM	poncho	DET	mosquito
		[Incorrect sentence] “The mosquito burnt the poncho.”						
B. SVO	a:	Ri	ch’utitata’	X-e-ru-pïs		ri	taq	lej
		DET	uncle	CP-ABS3pl-ERG3sg-wrap		DET	PM	tortilla
	b:	Ri	üs	x-e-ru-k’ät		ri	taq	nuq’u’
		DET	mosquito	CP-ABS3pl-ERG3sg-burn		DET	PM	poncho


The duration of each of the recorded, semantically plausible sentences was trimmed in Praat Version 5.3.53 (Boersma, 2001) to reduce the difference between the VOS and SVO sentences within each pair as much as possible. The difference between the duration of VOS sentences (*M* = 3456 ms, *SD* = 360) and that of SVO sentences (*M* = 3434 ms, *SD* = 347) was not significant [*t*(103) = 1.49, *p* = 0.14, *ns*.]. All the trimmed sentences were judged as natural in terms of prosody by our native Kaqchikel consultants.

### Procedure

The total number of stimuli (152) was divided between two sessions with 76 stimuli each. The stimuli were presented to participants in an event-related design with two sessions. Each session consisted of three conditions: verb-object-subject order (VOS), subject-verb-object order (SVO), and null task (N). The 152 stimuli were equally distributed across the two-task conditions (VOS and SVO), and there were 52 semantically plausible (“correct”) transitive sentences and 24 semantically implausible (“incorrect”) transitive sentences, except for the N condition, in which the participant made no response (**Table 1**). Before the scanning session, the experiment was explained outside the scanner and the participant practiced responding to shorter stimuli in a training session. During the experiment, individual participants wore headphones and stayed in supine positions inside the MRI scanner. Single sentence presentation did not exceed 5000 ms. The participants listened to the stimulus sentences in a random order through headphones. They were asked to judge whether each sentence was semantically plausible and to answer by pushing a YES button (correct sentence) or NO button (incorrect sentence), as quickly and accurately as possible. The duration between the beginning of each stimulus sentence and the button press was recorded as the reaction time (maximum = 8000 ms). All stimuli were controlled using E-prime (Psychology Software Tools).

### Image Acquisition

The data were acquired with a 3.0 Tesla MRI scanner (Philips Achieva Quasar Dual, Philips Medical Systems, Best, The Netherlands) while sentence comprehension tasks were conducted (VOS and SVO). Activation images were acquired using gradient-echo planer image (EPI) sequences with the following parameters: TE = 30 ms, field of view (FOV) = 192 mm, flip angle = 80°, slice thickness = 5 mm, slice gap = 0 mm. Twenty-five axial slices spanning the entire brain were obtained every 1.5 s. After the attainment of functional imaging, T1-weighted anatomical images were also acquired from each participant. We acquired 482 scans for each participant in each session.

### Analysis

All data processing and group analyses were performed using MATLAB (The Mathworks Inc., Natick, MA, USA) and SPM8 (Wellcome Department of Cognitive Neurology, London, UK). The acquisition timing of each slice was corrected using the middle (12th in time) slice as a reference for EPI data. In order to correct for head motion, functional images were first resliced and subsequently realigned with the first scan of the subjects. After alignment to the AC-PC line, each participant’s T1-weighted image was coregistered to the mean functional EPI image and segmented using the standard tissue probability maps provided in SPM8. Subsequently, realigned functional images were spatially normalized to the Montreal Neurological Institute (MNI) standard brain template, which converted voxel sizes to 3 mm × 3 mm × 3 mm, followed by smoothing using a Gaussian kernel with a full-width at half-maximum (FWHM) of 8 mm. An analysis of the tasks for each participant was conducted at the first statistical stage and group statistical analysis at the second stage. Contrasts between the VOS – SOV and SOV – VOS conditions were calculated using one sample *t*-test (*n* = 16) and masked by the VOS – N and SOV – N (significance threshold for masking was *p* < 0.05 uncorrected). The threshold for significant activation of each contrast was set at *p* < 0.05 (corrected for family wise error [FWE] rate). Finally, we performed a region of interest (ROI) analysis in the brain area obtained from the [SVO – VOS] comparison.

### Predictions

As noted above, the syntactically basic word order in Kaqchikel is VOS. SVO is a derived word order, although it is more frequently used than VOS. Given this, individual grammar theory would predict higher left IFG activation in the SVO condition than the VOS condition, whereas both universal cognition theory and usage-based theory would posit the opposite expectation.

## Results

### Behavioral Results

Statistical analyses were conducted using a linear mixed effects (LME) model. No significant differences were found either in accuracy rates for semantically plausible target sentences (*F*1, 1534 = 0.83, *p* = 0.36, *ns*.), or in reaction times for semantically plausible target sentences that were judged correctly (*F*1,1270 = 3.00, *p* = 0.08, *ns*.) (**Table 2**).

**Table 2:** Accuracy rates (%) and reaction times (ms) of sentence plausibility judgment for Kaqchikel VOS and SVO sentences.

	Accuracy rates (%)		Reaction times (ms)	
		
	M (%)	*SD* (%)	M (ms)	*SD* (ms)
VOS	83	37.7	4129	600
SVO	84	36.6	4081	587
	*p* = 0.36, *ns*		*p* = 0.08, *ns*	


**n* = 16.*

### Imaging Results

**FIGURE 1:**
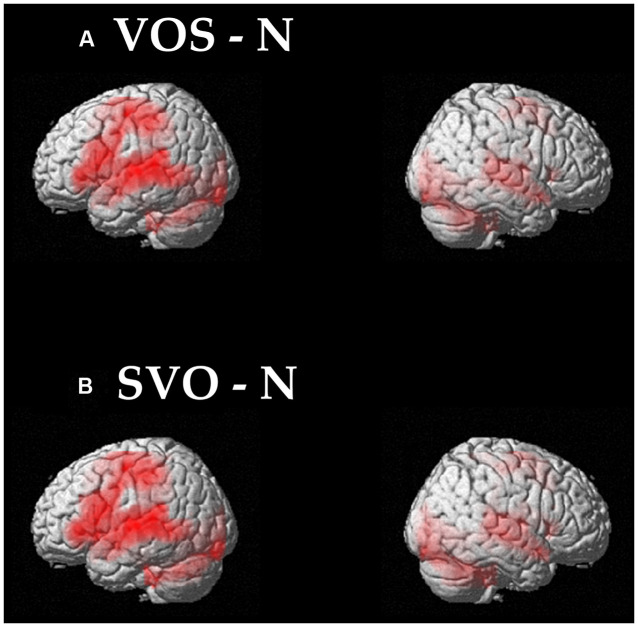
**Activated brain regions identified by comparisons between VOS – N (A) and SVO – N (B) conditions.** For display purposes, the threshold is set at *p* < 0.05 FDR, but the local maximum of the *t*-value reached a threshold of *p* < 0.05 FWE.

From the direct comparison between VOS – N and SVO – N, we identified the activated brain regions involved in the processing of VOS and SVO sentences, respectively. The results showed activations in the left inferior frontal gyrus and left middle temporal gyrus (**Table 3**; **Figure 1**). This suggests that most cognitive processes involved in the comprehension of SVO sentences are also involved in the comprehension of VOS sentences.

**Table 3:** Spatial coordinates of the local maxima in the group analysis.

Hemisphere	Anatomical region	MNI			*t*-value
			
		*x*	*y*	*z*	
**SVO > N**					
L	Superior temporal gyrus (BA 41)	-57	-19	4	9.34
L	Middle temporal gyrus (BA 22)	-60	-34	4	8.11
L	Superior temporal gyrus (BA 41)	-42	-25	10	7.57
L	Inferior frontal gyrus (BA 44)	-54	11	13	7.98
R	Superior temporal gyrus (BA 22)	51	-1	1	8.70
R	Superior temporal gyrus (BA 21)	54	-16	-2	7.49
L	Cerebellar tonsil	-6	-34	-47	8.50
R	Cerebellar tonsil	21	-52	-32	7.68
**VOS > N**					
L	Superior temporal gyrus (BA 41)	-57	-19	4	9.42
L	Superior temporal gyrus (BA 22)	-63	-34	13	8.34
L	Middle temporal gyrus (BA 22)	-60	-34	4	8.02
R	Superior temporal gyrus (BA 21)	54	-16	-2	8.22
L	Superior frontal gyrus (BA 6)	-9	11	49	8.95
R	Superior temporal gyrus (BA 22)	48	-34	7	7.66
L	Inferior frontal gyrus (BA 44)	-54	11	16	7.39
R	Superior frontal gyrus (BA 6)	3	-1	64	7.78
**VOS>SVO**					
no significant activation					
**SVO>VOS**					
L	Inferior frontal gyrus (BA 10/46)	-42	44	1	6.86


Coordinates are expressed in MNI space adopted by SPM8 in terms of distance (in mm) from the anterior commissure. The foci were anatomically localized on the standard stereotactic brain atlas developed by Talairach and Tournoux (1988), after correcting for differences between the MNI and Talairach coordinate systems using a nonlinear transformation. All statistical thresholds were set at corrected *p* < 0.05.

**FIGURE 2:**
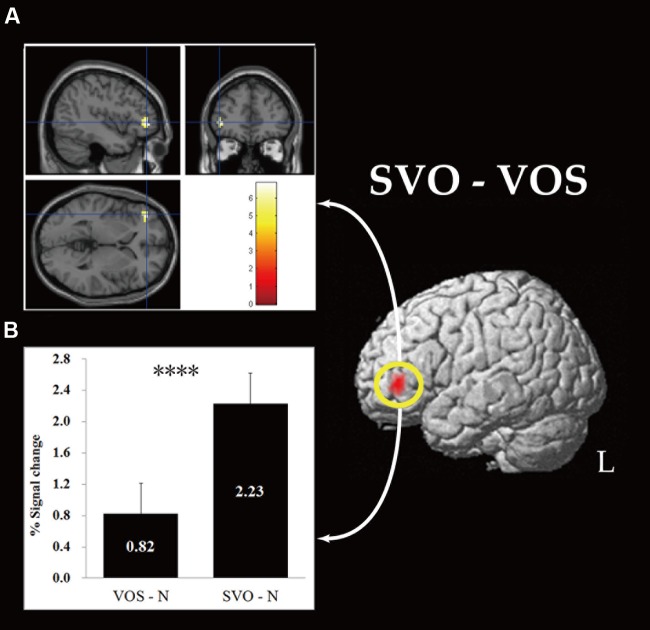
**Brain activation in MNI space **(A)** and ROI analysis **(B)** for the left IFG.** For display purposes, the threshold is set at *p* < 0.05 FDR, but the local maximum of the *t*-value reached a threshold of *p* < 0.05 FWE. ^∗∗∗∗^*p* < 0.001.

The direct comparison of data between SVO and VOS conditions showed cortical activation in the left inferior frontal gyrus close to the border with the left middle frontal gyrus [FWE, *p* < 0.05 (**Figure 2**)]. There was no significant activation in the reverse comparison.

## Discussion

The VOS word order of Kaqchikel allowed us to elucidate an unexplored aspect of sentence processing mechanisms. The higher left IFG activation in the SVO condition than in the VOS condition clearly shows that the grammatical features of the individual languages, not universal human cognitive features or production frequencies, primarily determine the sentence processing load. This is precisely what individual grammar theory predicts, but universal cognition theory or usage-based theory does not. Recall that universal cognition theory suggests that SO word orders should be easier to process than OS word orders, regardless of the basic word order of any individual language. Thus, it predicted that VOS should yield higher cortical activations than SVO in Kaqchikel, contrary to the experimental results reported above. Usage-based theory states that, other things being equal, the more frequent a construction is, the easier to process it should be. Therefore, this theory also led us to incorrectly expect larger cortical activations for VOS than SVO.

According to the psycholinguistic literature on SO languages, there are three major factors that are generally considered by individual grammar theory to contribute to the lower processing load of syntactically basic word orders compared to other grammatically possible orders: syntactic complexity, discourse-pragmatic requirements, and production frequency (Koizumi et al., 2014). First, the syntactically basic word order in a language, by definition, has simpler syntactic representations than the other grammatical orders in that language. Sentences with the syntactically basic word order are therefore easier to construct and access during sentence processing (Pritchett and Whitman, 1995; Gibson, 2000; Hawkins, 2004; Tamaoka et al., 2005). Second, the syntactically basic order can be felicitously used in a wider range of contexts, whereas derived orders require a specific discourse context. This discourse-pragmatic constraint for non-canonical word orders may be related to syntactic complexity: since non-canonical orders are associated with complex mental representations and hence are more difficult to process, speakers would bother to employ them only when necessary to achieve a specific goal. Thus, derived word orders incur higher processing loads when their discourse-pragmatic requirements are not met, for example, when presented out of context, as is the case in many processing experiments, including that of the present study (Kaiser and Trueswell, 2004; Weskott et al., 2011). Finally, the syntactically basic order tends to be more frequently used than other orders. The higher frequency of the syntactically basic word order may also be related to its syntactic complexity: since the syntactically basic word order is associated with simpler mental representations, other things being equal, it is easier to process than derived word orders. It therefore tends to be used more frequently. Since, other things being equal, more frequently used structures are processed faster and more accurately, the basic word order tends to be easier to process (Trueswell et al., 1993; MacDonald et al., 1994). In Japanese, for example, syntactically canonical SOV sentences have simpler syntactic structures than syntactically derived OSV sentences (Hoji, 1985; Saito, 1985). SOV sentences may be used in pragmatically neutral contexts, in contrast to OSV sentences, which are typically produced when the referent of the object is discourse-given (Kuno, 1978; Imamura and Koizumi, 2011; Koizumi and Imamura, 2016). The production frequency of SOV is higher than that of OSV (97.2 vs. 2.8%, respectively, according to Imamura and Koizumi, 2011). Together, these three factors seem to make SOV sentences easier to process than OSV sentences in Japanese (Koizumi and Imamura, 2016).

What is the case in Kaqchikel? As we have previously argued in Koizumi et al. (2014) and Yasunaga et al. (2015), in Kaqchikel, VOS is the syntactically basic word order, and therefore, it is associated with simpler syntactic structures than SVO or any other order. In terms of discourse-pragmatics, VOS can be used in various contexts, including a pragmatically neutral context. In contrast, SVO is preferentially employed when the subject is a topic (García Matzar et al., 1997; Ajsivinac Sian et al., 2004). Both of the syntactic and discourse-pragmatic factors presumably made the VOS sentences easier to process than the SVO sentences in the present experiment, which employed a sentence plausibility judgment task with no specific context provided. As for the relationship between processing load and word order frequency, however, Kaqchikel seems to be different from SO languages such as Japanese. As we have pointed out above, the production frequency of SVO is higher than that of VOS in Kaqchikel. The frequency factor, therefore, should facilitate the processing of SVO compared to VOS (= usage-based theory). The syntactic complexity and discourse-pragmatic factors, on the one hand, and the frequency of usage, on the other hand, presumably work in the opposite direction: the syntax and pragmatics favor VOS, whereas the frequency favors SVO. The former overwhelms the latter, resulting in the higher left IFG activation for SVO (cf. Bornkessel et al., 2002; Yasunaga et al., 2015). The discrimination between the effects of syntactic and discourse-pragmatic factors is beyond the scope of the present paper, and needs to be investigated in future research.

The discussion above leaves us with the question of why there is a discrepancy in Kaqchikel between the syntactically basic, easy to process word order, and the word order that is most frequently produced. This discrepancy might appear to be unique to OS languages such as Kaqchikel. However, we have previously argued in Koizumi et al. (2014) that if we shift our viewpoint slightly, similar situations can also be found in SO languages such as Japanese and Korean. In Japanese, for instance, the subject is marked with the nominative case marker when the sentence is used in pragmatically neutral contexts. When the referent of the subject is discourse-given and prominent, the subject is topicalized and marked with the topic marker (Kitagawa, 1982; Saito, 1985; Kuroda, 1988; Shibatani, 1990; Tateishi, 1990). This is schematically shown in example (4) (Shibatani, 1990).

a. [S-nom O V]
b. [S-top [ ____ O V]]

[S-nom OV] vs. [S-top OV] in Japanese seems to be comparable to VOS vs. SVO in Kaqchikel. On the one hand, Japanese [S-nom OV] and Kaqchikel VOS are both syntactically basic, and may be exploited in pragmatically neutral contexts. On the other hand, Japanese [S-top OV] and Kaqchikel SVO are syntactically more complex and employed in contexts where the subject is a discourse-topic (i.e., discourse-given information). In terms of the production frequencies, [S-top OV] and SVO are much higher than [S-nom OV] and VOS, respectively. It seems, therefore, that sentences with a topicalized subject tend to be associated with the information structure that creates cohesion among sentences and hence is most commonly used in natural discourse. They are thus more frequently employed than corresponding sentences with a non-topicalized subject in languages that grammatically distinguish between the two kinds of subjects. Viewed this way, it is quite natural that in Kaqchikel, SVO with a topicalized subject is produced more often than is VOS with a non-topicalized subject. It is therefore important to carefully examine the syntactic-pragmatic properties of the subject of SVO and VOS sentences in Kaqchikel in relation to their production frequencies, by comparing Kaqchikel, Japanese, and other languages including English, which do not morpho-syntactically distinguish between topicalized and non-topicalized subjects. We leave this task for future research.

## Conclusion

It has long been a matter of controversy whether subject-before-object word orders (SOV, SVO, VSO) are universally preferred to object-before-subject word orders (OSV, OVS, VOS) in language comprehension, production, and acquisition. To our knowledge, this paper presents the first neuroimaging data to show that, contrary to the common view that subject-before-object word orders are easier to process in any human language, an object-before-subject word order (VOS) is less demanding than other grammatically possible orders in at least one language, that is, the Kaqchikel Mayan language, whose syntactically basic word order is VOS. Based on this result, we argue that the SO preference in sentence comprehension may not reflect a universal aspect of human languages; rather, processing preference may be language-specific to some extent, reflecting syntactic differences in individual languages.

## Author Contributions

MK contributed to the experimental design. MK and JK created experimental materials and conducted the experiment. JK analyzed the data. MK and JK wrote the article. MK financially supported the study.

## Conflict of Interest Statement

The authors declare that the research was conducted in the absence of any commercial or financial relationships that could be construed as a potential conflict of interest.
